# 2-(2-Nitro­anilino)-4,5,6,7-tetra­hydro­benzo[*b*]thio­phene-3-carbonitrile

**DOI:** 10.1107/S1600536810035439

**Published:** 2010-09-11

**Authors:** Francisco J.B. Mendonça Junior, Maria do Carmo A. de Lima, Suely L. Galdino, Ivan R. Pitta, Carlos A. de Simone

**Affiliations:** a Laboratório de Síntese e Vetorização de Moléculas Bioativas, Universidade Estadual da Paraíba, 58020-540 João Pessoa, PB, Brazil; b Laboratório de Síntese e Planejamento de Fármacos, Departamento de Antibióticos, Universidade Federal de Pernambuco, 50670-910 Recife, PE, Brazil; c Departamento de Física e Informática, Instituto de Física de São Carlos, Universidade de São Paulo - USP, 13560-970 São Carlos, SP, Brazil

## Abstract

The title compound, C_15_H_13_N_3_O_2_S, was synthesized by the reaction of 2-amino-5,6,7,8-tetra­hydro-4*H*-cyclo­hepta­[*b*]thio­phene-3-carbonitrile and *o*-fluoro­nitro­benzene. The dihedral angle between the thio­phene and nitro­phenyl rings is 75.15 (2)°. In the crystal, inter­molecular N—H⋯N and C—H⋯O inter­actions lead to the formation of a supra­molecular chain extending along the *c*-axis direction.

## Related literature

For background to 2-substituted thio­phenes, see: Puterová *et al.* (2009 ▶). For the biological activity of 2-amino-benzo[*b*]thio­phene derivatives, see: Fakhr *et al.* (2008 ▶); Baraldi *et al.* (2006 ▶). For the synthesis of 2-amino thio­phenes, see: Gewald *et al.* (1966 ▶). For puckering parameters, see: Cremer & Pople (1975 ▶).
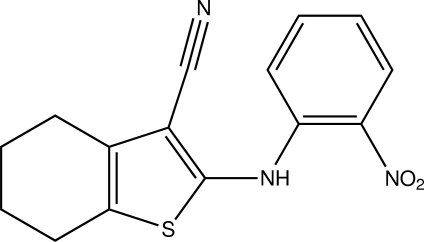


## Experimental

### 

#### Crystal data

C_15_H_13_N_3_O_2_S
*M* *_r_* = 299.34
Monoclinic, 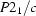

*a* = 13.2764 (4) Å
*b* = 13.4447 (7) Å
*c* = 8.2237 (4) Å
β = 106.794 (2)°
*V* = 1405.30 (11) Å^3^
*Z* = 4
Mo *K*α radiation
μ = 0.24 mm^−1^
*T* = 295 K
0.27 × 0.19 × 0.17 mm

#### Data collection

Nonius KappaCCD diffractometer
9590 measured reflections
3241 independent reflections
2351 reflections with *I* > 2σ(*I*)
*R*_int_ = 0.051

#### Refinement

*R*[*F* ^2^ > 2σ(*F* ^2^)] = 0.057
*wR*(*F* ^2^) = 0.174
*S* = 1.06
3241 reflections
192 parameters
H-atom parameters constrained
Δρ_max_ = 0.45 e Å^−3^
Δρ_min_ = −0.38 e Å^−3^

### 

Data collection: *COLLECT* (Nonius, 1997 ▶); cell refinement: *SCALEPACK* (Otwinowski & Minor, 1997 ▶); data reduction: *DENZO* (Otwinowski & Minor, 1997 ▶) and *SCALEPACK*; program(s) used to solve structure: *SHELXS97* (Sheldrick, 2008 ▶); program(s) used to refine structure: *SHELXL97* (Sheldrick, 2008 ▶); molecular graphics: *ORTEP-3 for Windows* (Farrugia, 1997 ▶); software used to prepare material for publication: *WinGX* (Farrugia, 1999 ▶).

## Supplementary Material

Crystal structure: contains datablocks I, global. DOI: 10.1107/S1600536810035439/tk2704sup1.cif

Structure factors: contains datablocks I. DOI: 10.1107/S1600536810035439/tk2704Isup2.hkl

Additional supplementary materials:  crystallographic information; 3D view; checkCIF report

## Figures and Tables

**Table 1:** Hydrogen-bond geometry (Å, °)

*D*—H⋯*A*	*D*—H	H⋯*A*	*D*⋯*A*	*D*—H⋯*A*
N2—H2⋯N1^i^	0.86	2.48	3.093 (3)	129
C11—H11⋯O1^ii^	0.93	2.56	3.351 (3)	143

Symmetry codes: (i) 
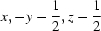
; (ii) 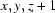.

**Fig. 1.**
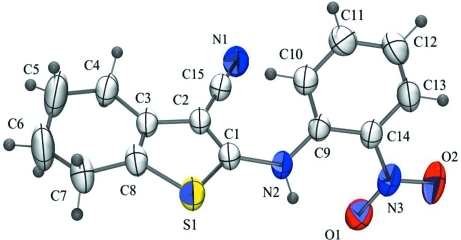
Projection of C15H13N3O2S, showing atom labelling and 50% probability displacement ellipsoids.

**Fig. 2.**
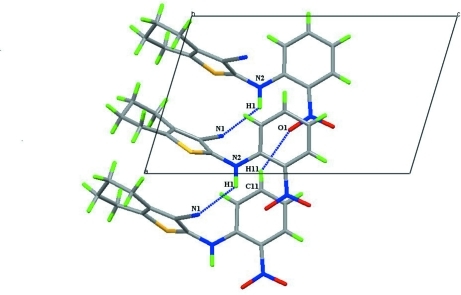
View of the packing along b axis showing intermolecular interactions as blue dashed lines.

## supplementary crystallographic information

### Comment

The various uses of 2-substituted thiophenes have been well documented (Puterová *et al.*, 2009). Amongst these applications, some 2-substituted benzo[*b*]thiophenes derivatives present anti-inflammatory and analgesic activities (Fakhr *et al.*, 2008), and others are adenosine A1 allosteric enhancers (Baraldi *et al.*, 2006). In this work, we report the structure of the title compound prepared by the reaction of 2-amino-5,6,7,8-tetrahydro-*4H*-cyclohepta[*b*]thiophene-3-carbonitrile and *o*-fluoro-nitrobenzene.

In the title compound, Fig. 1, the dihedral angle between least-squares planes passing through atoms of thiophene and nitrophenyl rings is 75.15 (2) °. The cyclohexane ring adopts a half-chair conformation with calculated puckering parameters of: q_2_ = 0.285 (5) Å, q_3_ = -0.240 (3) Å, Q_T_ = 0.373 (4) Å, θ = 130.2 (3) °, φ = -27.5 (6) ° (Cremer & Pople, 1975). In the packing, intermolecular N–H···N and C—H··· O interactions lead to the formation a supramolecular polymeric chain that extends along the *c* direction; Table 2 & Fig.2.

### Experimental

Under nitrogen and at 273 K, a dry THF solution (80 ml) of 2-amino-4,5,6,7- tetrahydro-*4H*-benzo[*b*]thiophene-3-carbonitrile (0.07 mol) and *o*-fluoro-nitrobenzene (0.07 mol) was added drop wise to a stirred suspension of NaH (0.105 mol) in dry THF (20 ml). The reaction mixture was stirred at room temperature for 24 h. The resulting mixture was adjusted to pH = 5 with 2 N HCl and then extracted with CHCl_3_. The extract was washed with aqueous Na_2_CO_3_ and water, dried over CaCl_2,_ and evaporated under reduced pressure. The dark-red solid obtained was purified by recrystallization from absolute ethanol, affording the title compound as red crystals; yield 11.72 g (56%), m.pt 275–276 K (Gewald *et al.*, 1966). Crystals were grown by evaporation at room temperature of its dichloromethane solution.

NMR ^1^H (200 MHz, CDCl_3_) δ: 1.84–1.87 (m, 4H), 2.63–2.73 (m, 4H), 6.91 (dt, 1H, *J* = 8.6, 1.4 Hz), 7.18 (dd, 1H, *J* = 8.6, 1.0 Hz), 7.51 (dt, 1H, *J* = 8.6, 8.2, 7.4 Hz), 8.22 (dd, 1H, *J* = 8.4, 1.4 Hz), 9.6 (s, 1H) p.p.m.

### Refinement

All H atoms attached were fixed geometrically and treated as riding with C—H = 0.93 Å (aromatic) or 0.97 Å (methylene) and N—H = 0.86 Å, and with *U*_iso_(H) = 1.2*U*_eq_(C or N).

### Crystal data

**Table:**

C_15_H_13_N_3_O_2_S	*F*(000) = 624
*M**_r_* = 299.34	*D*_x_ = 1.415 Mg m^−^^3^
Monoclinic, *P*2_1_/*c*	Mo *K*α radiation, λ = 0.71073 Å
Hall symbol: -P 2ybc	Cell parameters from 4953 reflections
*a* = 13.2764 (4) Å	θ = 2.9–27.5°
*b* = 13.4447 (7) Å	µ = 0.24 mm^−^^1^
*c* = 8.2237 (4) Å	*T* = 295 K
β = 106.794 (2)°	Prism, yellow
*V* = 1405.30 (11) Å^3^	0.27 × 0.19 × 0.17 mm
*Z* = 4	

### Data collection

**Table:**

Nonius KappaCCD diffractometer	2351 reflections with *I* > 2σ(*I*)
Radiation source: Enraf Nonius FR590	*R*_int_ = 0.051
horizonally mounted graphite crystal	θ_max_ = 27.5°, θ_min_ = 3.0°
Detector resolution: 9 pixels mm^-1^	*h* = −17→17
CCD rotation images,thick slices scans	*k* = −17→17
9590 measured reflections	*l* = −9→10
3241 independent reflections	

### Refinement

**Table:**

Refinement on *F*^2^	Secondary atom site location: difference Fourier map
Least-squares matrix: full	Hydrogen site location: inferred from neighbouring sites
*R*[*F*^2^ > 2σ(*F*^2^)] = 0.057	H-atom parameters constrained
*wR*(*F*^2^) = 0.174	*w* = 1/[σ^2^(*F*_o_^2^) + (0.1015*P*)^2^ + 0.2662*P*] where *P* = (*F*_o_^2^ + 2*F*_c_^2^)/3
*S* = 1.06	(Δ/σ)_max_ < 0.001
3241 reflections	Δρ_max_ = 0.45 e Å^−^^3^
192 parameters	Δρ_min_ = −0.38 e Å^−^^3^
0 restraints	Extinction correction: *SHELXL97* (Sheldrick, 2008), Fc^*^=kFc[1+0.001xFc^2^λ^3^/sin(2θ)]^-1/4^
Primary atom site location: structure-invariant direct methods	Extinction coefficient: 0.133 (13)

### Special details

**Table:**

Geometry. All e.s.d.'s (except the e.s.d. in the dihedral angle between two l.s. planes) are estimated using the full covariance matrix. The cell e.s.d.'s are taken into account individually in the estimation of e.s.d.'s in distances, angles and torsion angles; correlations between e.s.d.'s in cell parameters are only used when they are defined by crystal symmetry. An approximate (isotropic) treatment of cell e.s.d.'s is used for estimating e.s.d.'s involving l.s. planes.
Refinement. Refinement of *F*^2^ against ALL reflections. The weighted *R*-factor *wR* and goodness of fit *S* are based on *F*^2^, conventional *R*-factors *R* are based on *F*, with *F* set to zero for negative *F*^2^. The threshold expression of *F*^2^ > σ(*F*^2^) is used only for calculating *R*-factors(gt) *etc*. and is not relevant to the choice of reflections for refinement. *R*-factors based on *F*^2^ are statistically about twice as large as those based on *F*, and *R*- factors based on ALL data will be even larger.

### Fractional atomic coordinates and isotropic or equivalent isotropic displacement parameters (Å^2^)

**Table:**

	*x*	*y*	*z*	*U*_iso_*/*U*_eq_	
C1	−0.22931 (15)	−0.11377 (16)	−0.8690 (3)	0.0489 (5)	
C2	−0.17013 (15)	−0.19140 (16)	−0.7859 (2)	0.0470 (5)	
C3	−0.06070 (15)	−0.16847 (17)	−0.7171 (3)	0.0494 (5)	
C4	0.02377 (17)	−0.2391 (2)	−0.6205 (3)	0.0620 (6)	
H4A	0.0009	−0.2717	−0.5322	0.074*	
H4B	0.0352	−0.2898	−0.6972	0.074*	
C5	0.1254 (2)	−0.1846 (3)	−0.5417 (5)	0.1058 (12)	
H5A	0.1231	−0.1586	−0.4328	0.127*	
H5B	0.1823	−0.2325	−0.5196	0.127*	
C6	0.1505 (2)	−0.1041 (3)	−0.6378 (6)	0.1056 (13)	
H6A	0.1667	−0.1319	−0.7362	0.127*	
H6B	0.2138	−0.0720	−0.5686	0.127*	
C7	0.06661 (19)	−0.0251 (2)	−0.6990 (4)	0.0700 (7)	
H7A	0.0705	0.0225	−0.6088	0.084*	
H7B	0.0783	0.0102	−0.7948	0.084*	
C8	−0.04015 (15)	−0.07297 (18)	−0.7510 (3)	0.0540 (5)	
C9	−0.41366 (15)	−0.11662 (14)	−0.8689 (3)	0.0449 (5)	
C10	−0.38494 (18)	−0.10942 (18)	−0.6915 (3)	0.0560 (6)	
H10	−0.3142	−0.1031	−0.6314	0.067*	
C11	−0.4588 (2)	−0.1114 (2)	−0.6043 (3)	0.0649 (6)	
H11	−0.4374	−0.1062	−0.4865	0.078*	
C12	−0.5649 (2)	−0.1210 (2)	−0.6897 (4)	0.0703 (7)	
H12	−0.6145	−0.1229	−0.6298	0.084*	
C13	−0.59545 (18)	−0.12779 (18)	−0.8620 (4)	0.0622 (6)	
H13	−0.6665	−0.1338	−0.9202	0.075*	
C14	−0.52138 (16)	−0.12570 (15)	−0.9526 (3)	0.0486 (5)	
C15	−0.21465 (15)	−0.28666 (18)	−0.7727 (3)	0.0519 (5)	
N1	−0.25004 (17)	−0.36281 (16)	−0.7606 (3)	0.0673 (6)	
N2	−0.33714 (13)	−0.11309 (15)	−0.9523 (2)	0.0524 (5)	
H2	−0.3569	−0.1103	−1.0614	0.063*	
N3	−0.56173 (15)	−0.13233 (14)	−1.1361 (3)	0.0572 (5)	
O1	−0.49971 (14)	−0.12880 (15)	−1.2218 (2)	0.0713 (5)	
O2	−0.65641 (14)	−0.14080 (17)	−1.2012 (3)	0.0866 (6)	
S1	−0.15219 (4)	−0.01029 (5)	−0.86321 (8)	0.0610 (3)	

### Atomic displacement parameters (Å^2^)

**Table:**

	*U*^11^	*U*^22^	*U*^33^	*U*^12^	*U*^13^	*U*^23^
C1	0.0350 (10)	0.0626 (12)	0.0490 (11)	0.0004 (8)	0.0119 (8)	0.0021 (9)
C2	0.0348 (9)	0.0602 (12)	0.0452 (10)	0.0001 (8)	0.0105 (8)	0.0005 (9)
C3	0.0342 (10)	0.0649 (12)	0.0479 (10)	0.0015 (8)	0.0103 (8)	−0.0039 (9)
C4	0.0414 (11)	0.0756 (15)	0.0637 (13)	0.0065 (10)	0.0066 (10)	0.0019 (12)
C5	0.0452 (15)	0.113 (3)	0.134 (3)	0.0006 (15)	−0.0129 (17)	0.017 (2)
C6	0.0401 (14)	0.111 (3)	0.155 (4)	−0.0094 (14)	0.0110 (17)	0.012 (2)
C7	0.0415 (11)	0.0811 (17)	0.0843 (17)	−0.0116 (11)	0.0133 (11)	−0.0086 (14)
C8	0.0371 (10)	0.0652 (13)	0.0598 (12)	−0.0011 (9)	0.0140 (9)	−0.0044 (10)
C9	0.0365 (9)	0.0465 (10)	0.0504 (10)	0.0035 (7)	0.0104 (8)	0.0042 (8)
C10	0.0433 (11)	0.0699 (14)	0.0537 (12)	0.0050 (10)	0.0123 (9)	0.0017 (10)
C11	0.0588 (14)	0.0817 (17)	0.0590 (13)	0.0141 (12)	0.0246 (11)	0.0063 (12)
C12	0.0552 (14)	0.0834 (18)	0.0825 (18)	0.0101 (12)	0.0363 (13)	0.0121 (14)
C13	0.0391 (11)	0.0643 (14)	0.0836 (17)	0.0018 (9)	0.0182 (11)	0.0075 (12)
C14	0.0375 (10)	0.0461 (10)	0.0590 (12)	0.0030 (8)	0.0088 (9)	0.0038 (9)
C15	0.0373 (10)	0.0654 (13)	0.0502 (11)	0.0030 (9)	0.0081 (8)	0.0052 (10)
N1	0.0534 (11)	0.0684 (13)	0.0759 (13)	−0.0033 (10)	0.0121 (10)	0.0102 (10)
N2	0.0335 (8)	0.0765 (12)	0.0444 (9)	0.0030 (8)	0.0069 (7)	0.0035 (8)
N3	0.0420 (9)	0.0574 (11)	0.0631 (11)	0.0020 (8)	0.0010 (8)	−0.0018 (9)
O1	0.0580 (10)	0.0953 (14)	0.0540 (9)	−0.0013 (9)	0.0056 (8)	0.0017 (9)
O2	0.0413 (9)	0.1135 (16)	0.0871 (13)	0.0028 (9)	−0.0099 (9)	−0.0141 (11)
S1	0.0445 (4)	0.0594 (4)	0.0757 (5)	−0.0014 (2)	0.0119 (3)	0.0052 (3)

### Geometric parameters (Å, °)

**Table:**

C1—C2	1.365 (3)	C7—H7B	0.9700
C1—N2	1.397 (2)	C8—S1	1.727 (2)
C1—S1	1.720 (2)	C9—N2	1.381 (2)
C2—C15	1.428 (3)	C9—C10	1.401 (3)
C2—C3	1.432 (3)	C9—C14	1.402 (3)
C3—C8	1.358 (3)	C10—C11	1.372 (3)
C3—C4	1.508 (3)	C10—H10	0.9300
C4—C5	1.507 (4)	C11—C12	1.386 (4)
C4—H4A	0.9700	C11—H11	0.9300
C4—H4B	0.9700	C12—C13	1.360 (4)
C5—C6	1.436 (5)	C12—H12	0.9300
C5—H5A	0.9700	C13—C14	1.395 (3)
C5—H5B	0.9700	C13—H13	0.9300
C6—C7	1.516 (4)	C14—N3	1.451 (3)
C6—H6A	0.9700	C15—N1	1.143 (3)
C6—H6B	0.9700	N2—H2	0.8600
C7—C8	1.502 (3)	N3—O2	1.221 (3)
C7—H7A	0.9700	N3—O1	1.230 (3)
			
C2—C1—N2	127.62 (19)	C6—C7—H7B	109.7
C2—C1—S1	110.65 (15)	H7A—C7—H7B	108.2
N2—C1—S1	121.72 (16)	C3—C8—C7	125.1 (2)
C1—C2—C15	122.20 (18)	C3—C8—S1	112.21 (15)
C1—C2—C3	113.87 (19)	C7—C8—S1	122.6 (2)
C15—C2—C3	123.92 (19)	N2—C9—C10	119.80 (18)
C8—C3—C2	111.30 (19)	N2—C9—C14	123.48 (19)
C8—C3—C4	122.69 (19)	C10—C9—C14	116.71 (19)
C2—C3—C4	126.0 (2)	C11—C10—C9	121.5 (2)
C5—C4—C3	111.0 (2)	C11—C10—H10	119.2
C5—C4—H4A	109.4	C9—C10—H10	119.2
C3—C4—H4A	109.4	C10—C11—C12	120.8 (2)
C5—C4—H4B	109.4	C10—C11—H11	119.6
C3—C4—H4B	109.4	C12—C11—H11	119.6
H4A—C4—H4B	108.0	C13—C12—C11	119.2 (2)
C6—C5—C4	116.8 (3)	C13—C12—H12	120.4
C6—C5—H5A	108.1	C11—C12—H12	120.4
C4—C5—H5A	108.1	C12—C13—C14	120.7 (2)
C6—C5—H5B	108.1	C12—C13—H13	119.6
C4—C5—H5B	108.1	C14—C13—H13	119.6
H5A—C5—H5B	107.3	C13—C14—C9	121.1 (2)
C5—C6—C7	116.4 (3)	C13—C14—N3	116.7 (2)
C5—C6—H6A	108.2	C9—C14—N3	122.25 (19)
C7—C6—H6A	108.2	N1—C15—C2	179.4 (2)
C5—C6—H6B	108.2	C9—N2—C1	123.57 (17)
C7—C6—H6B	108.2	C9—N2—H2	118.2
H6A—C6—H6B	107.3	C1—N2—H2	118.2
C8—C7—C6	109.7 (2)	O2—N3—O1	121.8 (2)
C8—C7—H7A	109.7	O2—N3—C14	119.0 (2)
C6—C7—H7A	109.7	O1—N3—C14	119.12 (18)
C8—C7—H7B	109.7	C1—S1—C8	91.95 (10)

### Hydrogen-bond geometry (Å, °)

**Table:**

*D*—H···*A*	*D*—H	H···*A*	*D*···*A*	*D*—H···*A*
N2—H2···N1^i^	0.86	2.48	3.093 (3)	129
C11—H11···O1^ii^	0.93	2.56	3.351 (3)	143

Symmetry codes: (i) *x*, −*y*−1/2, *z*−1/2; (ii) *x*, *y*, *z*+1.
